# Influence of Free Radicals Signal from Dental Resins on the Radio-Induced Signal in Teeth in EPR Retrospective Dosimetry

**DOI:** 10.1371/journal.pone.0062225

**Published:** 2013-05-21

**Authors:** Philippe Levêque, Céline Desmet, Ana Maria Dos Santos-Goncalvez, Sébastien Beun, Julian G. Leprince, Gaëtane Leloup, Bernard Gallez

**Affiliations:** 1 Biomedical Magnetic Resonance Research Group, Louvain Drug Research Institute, Université catholique de Louvain, Brussels, Belgium; 2 Center for Research and Engineering on Biomaterials CRIBIO, Université catholique de Louvain, Brussels, Belgium; 3 Institute of Condensed Matter and Nanosciences, Bio- and Soft- Matter, Université catholique de Louvain, Louvain-la-Neuve, Belgium; 4 School of Dentistry and Stomatology, Université catholique de Louvain, Brussels, Belgium; University of Zurich, Switzerland

## Abstract

In case of radiological accident, retrospective dosimetry is needed to reconstruct the absorbed dose of overexposed individuals not wearing personal dosimeters at the onset of the incident. In such a situation, emergency mass triage will be required. In this context, it has been shown that Electron Paramagnetic Resonance (EPR) spectroscopy would be a rapid and sensitive method, on the field deployable system, allowing dose evaluation of a great number of people in a short time period. This methodology uses tooth enamel as a natural dosimeter. Ionising radiations create stable free radicals in the enamel, in a dose dependent manner, which can be detected by EPR directly in the mouth with an appropriate resonator. Teeth are often subject to restorations, currently made of synthetic dimethacrylate-based photopolymerizable composites. It is known that some dental composites give an EPR signal which is likely to interfere with the dosimetric signal from the enamel. So far, no information was available about the occurrence of this signal in the various composites available on the market, the magnitude of the signal compared to the dosimetric signal, nor its evolution with time. In this study, we conducted a systematic characterization of the signal (intensity, kinetics, interference with dosimetric signal) on 19 most widely used composites for tooth restoration, and on 14 experimental resins made with the most characteristic monomers found in commercial composites. Although a strong EPR signal was observed in every material, a rapid decay of the signal was noted. Six months after the polymerization, the signal was negligible in most composites compared to a 3 Gy dosimetric signal in a tooth. In some cases, a stable atypical signal was observed, which was still interfering with the dosimetric signal.

## Introduction

In the aftermath of a major radiological incident involving several thousands of individuals, such as a terrorist attack with a dirty bomb in the transportation system of a large city, or a major nuclear plant accident, the various health institutions and emergency services would face a dramatic increase in victims requiring medical attention, and would most probably be overwhelmed by this huge number of victims to treat, not to mention worried people not necessitating health care [1], [2].

Hence, there is a critical need for mass triage strategies determining the absorbed dose, which is a key parameter for appropriate medical treatment [3]. Without appropriate treatment, nearly all individuals exposed to more than 4 Gy would die within 30 days [4]–[6].

Several programs have been initiated aiming at developing adequate tools that would allow rapid and efficient dose estimation, preferably on the field, of a large number of individuals not equipped with conventional dosimeter [7]–[11]. Electron Paramagnetic Resonance (EPR) spectroscopy, using dental enamel as a natural dosimeter, appeared to have significant advantages over other dose assessment techniques, such as biological assays or mathematical dose reconstructing using MonteCarlo simulations [12], [13]. EPR provides quick measurements, and is deployable on the field. Several dosimetric materials usually found on victims have been proposed, among which tooth enamel is the most promising [14]. Ionising radiations induce the formation of stable free radicals, mostly CO_2_^−^, in tooth enamel, the external layer of tooth, mainly composed of hydroxyapatite (Ca_10_(PO_4_)_6_(OH)_2_).

These free radicals are detected and quantified by EPR spectroscopy [15]. EPR spectrometers have been recently adapted for non invasive measurements directly in the mouth of a patient, and on the field deployable systems have been developed by the pioneer work of the Dartmouth Center for Medical Countermeasures (CMCR) [16]–[18].

Some issues remain to be solved before full validation of the technique. One remaining question is the influence of teeth restorations on the dosimetric signal [19]. Indeed, in the western population, teeth are commonly subject to restorations, most often made of synthetic resin-based photopolymerizable composites [20]. Composites used in dental practice are composed of inorganic fillers (from 50% to 80% w/w), dispersed in an organic matrix (from 20% to 50% w/w) [21]. The organic matrix comprises various proportions of methacrylic monomers, a photoinitiator, e. g. camphorquinone, and a co-initiator, e. g. a tertiary amine [22]. High intensity visible light will trigger the polymerization reaction, which is a free-radical mediated reaction. As the reaction progresses, connections between the dimethacrylate monomers are established and a three-dimensional polymer network is generated. The material becomes harder; at the end of the process, most radicals will recombine, but some of them will not because of the vitrification process which diminishes the mobility of the molecules in the material. Free radicals remain trapped in the matrix and can be detected by EPR. Two types of free radicals are known to be generated, their spectrum being superimposed to give a complex and broad multilines spectrum [23]. The spectrum is complex because of the hyperfine splitting, and some lines are unfortunately positioned at the same frequency as the dosimetric signal of the enamel so that interferences between those signals are likely to occur. This would consequently lead to an overestimation of the measured dose. So far, no information was available about the occurrence of this signal in the various composites available on the market, the magnitude of the signal compared to the dosimetric signal, nor its evolution with time.

In this study, we conducted a systematic characterization of the signal on 19 commercial composites among the most widely used materials for tooth restoration, and on 14 experimental resins made with the monomers usually found in commercial composites for a possible class effect. Indeed monomers found in commercial composites are usually bisphenol A glycidyl dimethacrylate (Bis-GMA), triethylene glycol dimethacrylate (TEGDMA), urethane dimethacrylate (UDMA) or bisphenol A ethoxylate dimethacrylates (Bis-EMA) in different proportions. Signal intensity, kinetics and interference with dosimetric signal were studied in X-band mode for sensitivity and in L-band mode, which is the mode used for non-invasive measurements in human. This study lasted for six months and involved over 3000 measurements.

## Materials and Methods

### Composites

Among the commercial composites the most widely used by dentists, 19 were selected for this study because they are routinely used for restoration of incisors (table 1).

**Table 1:** Commercial composites selected for this study among the most widely used composites on the market.

Composite	Shade	Lot	Brand
Filtek Supreme Ultra	A3	N265426	3M-ESPE, St Paul, MN, USA
Venus Diamond	A3	010040	Heraeus-Kulzer, Wehrheim, Germany
IPS Empress Direct	A3	P02374	Ivoclar-Vivadent, Schaan, Liechtenstein
Tetric EvoCeram	A3	P11989	Ivoclar-Vivadent, Schaan, Liechtenstein
Amaris	O3	1121316	Voco GmbH, Cuxhaven, Germany
GrandioSo	A3	1120117	Voco GmbH, Cuxhaven, Germany
Gradia Direct X	A3	1103081	GC Europe N.V., Leuven, Belgium
GC Kalore	A3	1007201	GC Europe N.V., Leuven, Belgium
Ice	A3	110150T	Southern Dental Industries, Australia
N'Durance	A3	11011OB	Septodont, Saint-Maur-des-Fossés, France
Clearfil AP-X	A3	1383AA	Kuraray Europe GmbH, Hattersheim am Main, Germany
Clearfil Majesty Esthetic	A3	0038CA	Kuraray Europe GmbH, Hattersheim am Main, Germany
Synergy D6	A3	C42276	Coltène-Whaledent, Langenau, Germany
Esthet-X HD	A3	1106102	Dentsply Caulk, Milford, DE, USA
TPH3	A3	1110000495	Dentsply Caulk, Milford, DE, USA
Ceram-X	A3	1110000028	Dentsply Caulk, Milford, DE, USA
Artiste Nano	A3	3666316	Pentron Clinical, Orange, CA, USA
Simile	A3	4328025	Pentron Clinical, Orange, CA, USA
Herculite Ultra	A3	3978906	Kerr Corporation, Orange, CA, USA

Experimental resins were prepared using different proportions of the most common monomers, in order to investigate for a possible class effect.

Bisphenol A glycidyl dimethacrylate (Bis-GMA), triethylene glycol dimethacrylate (TEGDMA), urethane dimethacrylate (UDMA), ethoxylated bisphenol A glycidyl dimethacrylate (Bis-EMA-2 and Bis-EMA-15) were all purchased from Sigma-Aldrich (Belgium). Fourteen compositions of these monomers were prepared (table 2) according to Sideridou et al. [24]. Each composition contained 2% (molar) camphorquinone (Sigma-Aldrich, Belgium) as the photo-initiator and 2% (molar) ethyl-4-dimethylaminobenzoate (Sigma-Aldrich, Belgium) as the co-initiator.

**Table 2:** Composition of experimental resins.

Monomers	Molar ratio	%weight
G	1	100
T	1	100
U	1	100
E-15	1	100
E-2	1	100
G/T	0.3582/0.6418	50/50
G/T	0.5659/0.4341	70/30
G/U	0.3582/0.6418	37.8/62.2
G/U	0.5659/0.4341	58.7/41.3
G/E-15	0.3582/0.6418	43.2/56.8
G/E-15	0.5659/0.4341	64/36
G/T/U/E-15	0.5660/0.2340/0.1/0.1	65.7/15.2/10.6/8.5
G/T/U/E-15	0.5660/0.1340/0.15/0.15	63.7/8.4/15.5/12.4
G/U/E-15	0.5660/0.2170/0.2170	61.3/21.5/17.2

G: Bis-GMA, T: TEGDMA, U: UDMA, E-15: Bis-EMA (15-ethoxy/phenol), E-2: Bis-EMA (2-ethoxy/phenol).

### Polymerization of samples

Resins were polymerized in a PTFE mould (7 mm×1.4 mm×1.4 mm) using a BluePhase G2 lamp (Ivoclar Vivadent, Schaan, Liechtenstein) at high power (1200 mW/cm^2^) during 20 seconds. The mass of the sample was 30 mg, a typical medium size restoration on an incisor. Power was regularly checked with a radiometer. The lamp was placed reproducibly in a fixed position at 5 mm from the mould. Five samples were photopolymerized for each composite, both for commercial and experimental resins. Samples were stored in dry conditions and in the dark during all the study.

### Dental enamel powder

Dental enamel powder was obtained by crushing the crown of a molar tooth after removal of the dentin. The molar was obtained from the collection of the laboratory of anatomy (practical courses) at the faculty of medicine, Université catholique de Louvain. The powder was irradiated at 3 and 10 Gy (absorbed dose in water) with a ^137^Cs gamma irradiator (IBL637, Oris Industrie). The dosimetric signal was measured in X-band with the same settings than those used for dental resins.

### Ethics statement

This study was approved by the local ethics committee “Commission d'éthique biomédicale hospitalo-facultaire” from the university hospital “Cliniques universitaires Saint-Luc” (IRB 00001530).

### EPR measurements

#### X band

X-band measurements were recorded with a Miniscope MS200 spectrometer (Magnettech, Berlin, Germany) operating at ∼9.5 GHz. The spectrometer was calibrated with a standard of 2,2-diphenyl-1-picrylhydrazyl (dpph) (Bruker Biospin, Rheinstetten, Germany) each time before and after each series of measurements.

The acquisition parameters were as follows: center field: 335.60 mT, sweep field: 12.99 mT, acquisition time: 30 s, smooth: 0, number of points: 512, number of scans: 1, modulation frequency: 100 kHz, modulation amplitude: 0.1 mT, power: 0.5 mW, gain: 30.

The intensity of the signal was measured as the peak-to-peak height of the central peak of the spectrum. This intensity was normalized with the signal intensity of the dpph standard and expressed as normalized units (n.u.).

#### L band

Measurements were recorded with a L-band spectrometer (Magnettech, Berlin, Germany) operating at ∼1.2 GHz, with a surface coil resonator (diameter 10 mm). Samples were placed reproducibly in the loop of the resonator. The spectrometer was calibrated with a standard of dpph (Alpha Caesar, diluted 1/28 in sucrose) each time before and after each series of measurements.

Recordings were performed with the following settings: center field: 57 mT, sweep field: 10 mT, acquisition time: 30 s, smooth: 0.50, number of points: 512, number of scans: 5, modulation frequency: 100 kHz, modulation amplitude: 0.375 mT, power: 15 mW, gain: 200. For late measurements, the number of scans was increased to 50.

The signal intensity was measured as the peak-to-peak height of the central peak of the spectrum. This intensity was normalized with the signal intensity of the dpph and expressed as normalized units (n. u.).

### Kinetics of decay

In order to follow the signal decay over a long period of time (i.e. months), samples were measured using the EPR most sensitive mode, namely X-band at 9.5 GHz. Measurements were taken 5 minutes and 1 hour after completion of the photopolymerization, repeated once a day during the first week, then on two consecutive days per week during the first month, and finally on two consecutive days per month for the remaining five months.

Samples were also measured in L-band mode, in order to determine the limit of detection of this low sensitivity mode, but compatible with *in vivo* measurements.

Measurements were performed 65 minutes after photopolymerization, then once a day during the first week, repeated on two consecutive days per week during the first month, and finally on two consecutive days per month for last five months.

Curves were fitted with a bi-exponential decay model using Prism 5 (GraphPad Software, La Jolla, CA, USA). Where the bi-exponential model was no statistically significant, a mono-exponential model was used.

## Results

### Screening of initial EPR signal

**Figure 1:**
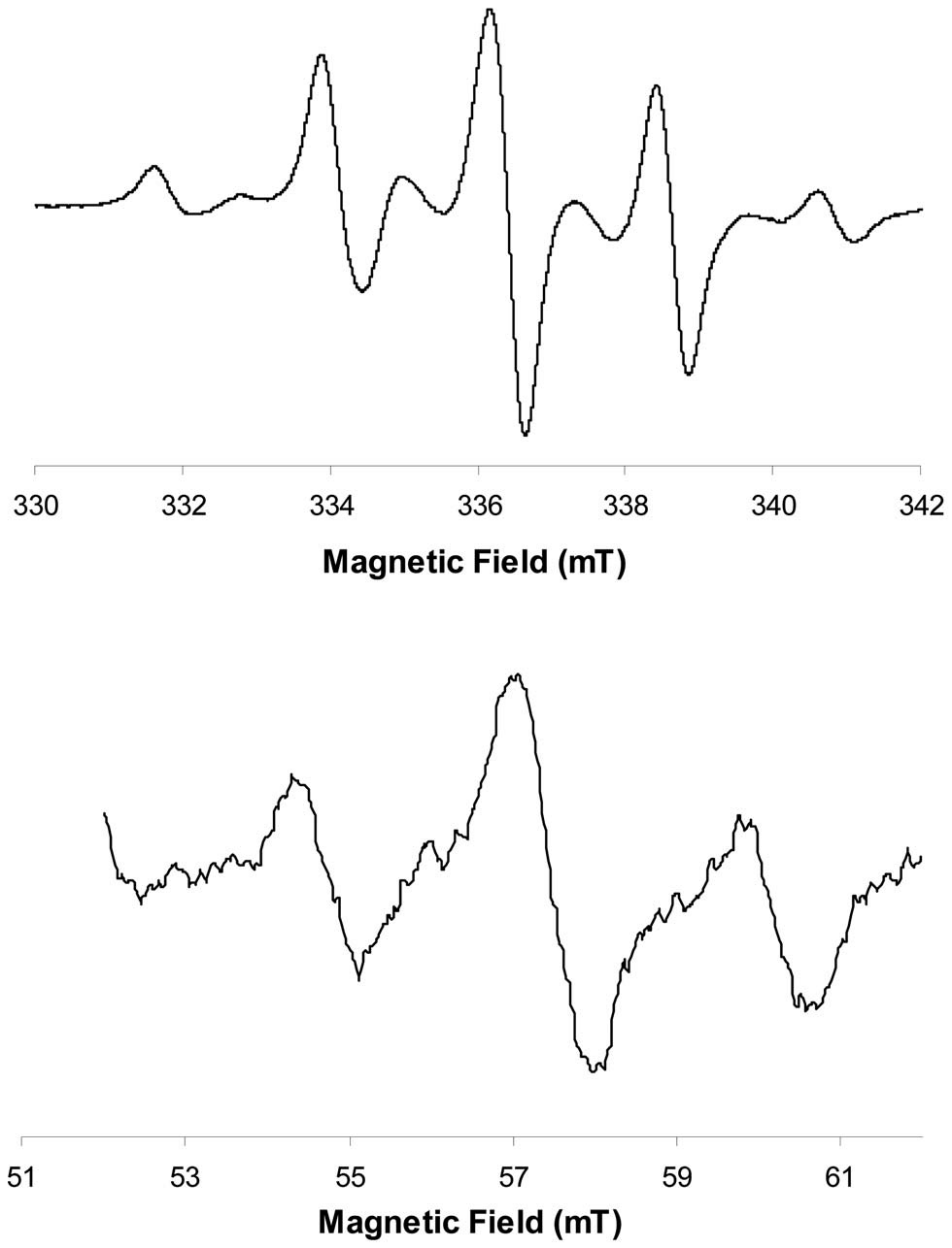
Typical 9 lines EPR spectrum observed in X-band (top) and L-band (bottom) for a commercial composite (Filtek Supreme Ultra).

**Figure 2:**
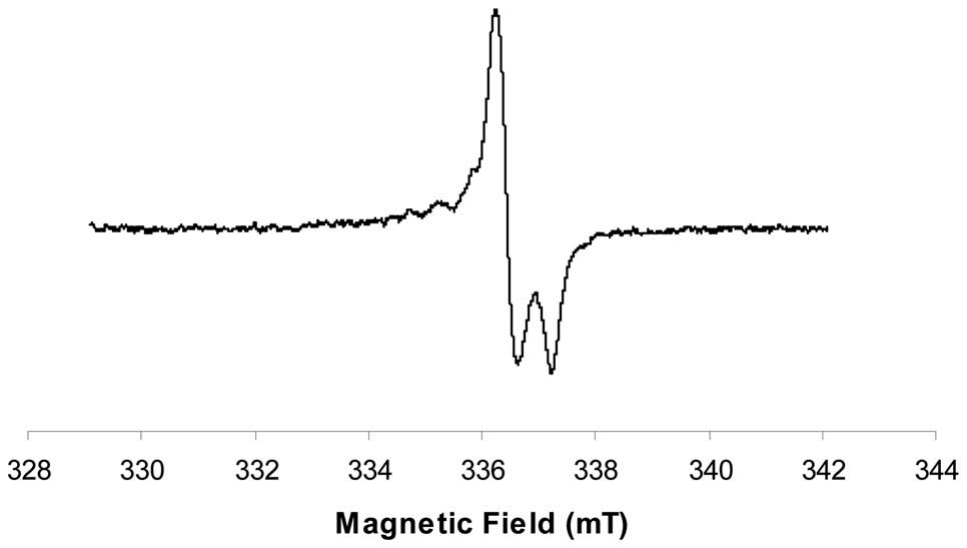
EPR dosimetric signal induced by radiations in tooth enamel.

**Figure 3:**
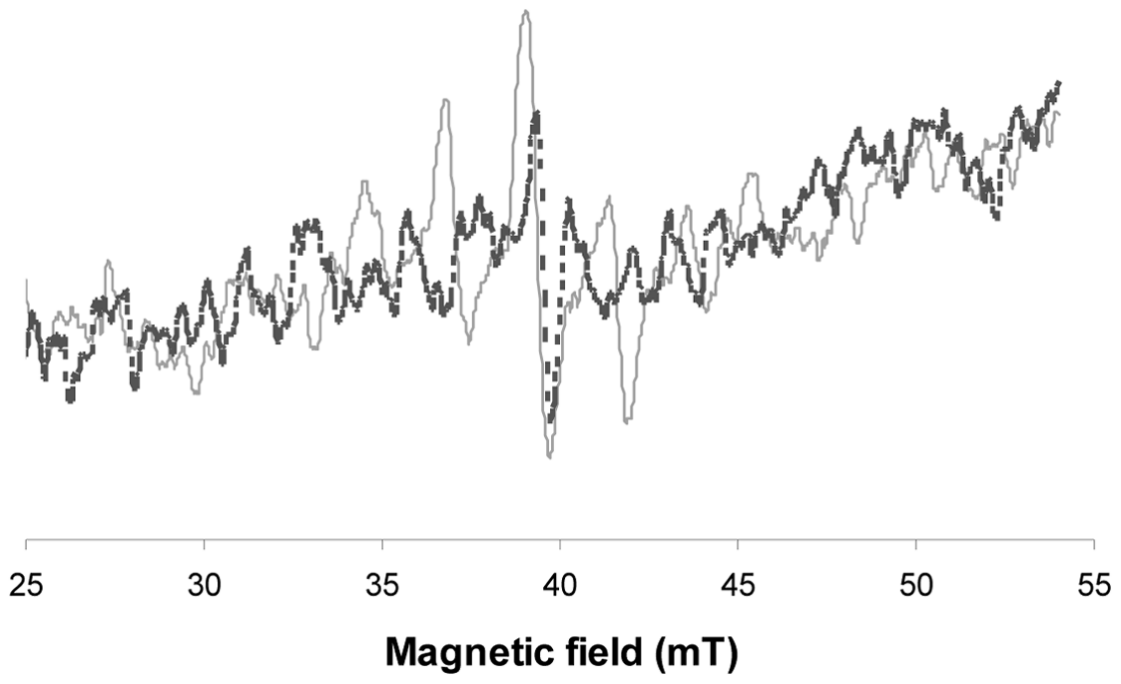
(dotted line) L-band EPR spectrum of an unrestored irradiated tooth (10Gy) and (plain line) restored and irradiated tooth (10Gy).

A strong EPR signal with a typical nine lines spectrum was observed in all the composites tested, commercial as well as experimental ones (Fig. 1). This complex spectrum is due to the presence of two radical species, an allylic radical and a propagating radical [23]. The central line is positioned at the same resonance frequency as the dosimetric signal in tooth enamel (Fig. 2 & 3). Shortly after the initiation of the polymerization by light, the signal in commercial composites was very high, ranging from 6.0 n.u. for the Venus Diamond composite, to 38.3 n.u. for the N'Durance (table 3, median  = 16.0 n.u.). This is 16.5 to 106.4 higher than the signal recorded for a tooth irradiated at 3 Gy (0.3 n.u.).

**Table 3:** Normalized intensities of EPR signal recorded in commercial resins 5 minutes after polymerization (X-band), and 65 minutes after polymerization (L-band).

Commercial composites	Intensity 5 min X-Band Normalized units ± sem (n = 5)	Intensity 65 min L-Band Normalized units ± sem (n = 5)
Filtek Supreme Ultra	34.5±1.2	0.62±0.05
Venus Diamond	6.0±0.2	0.18±0.03
IPS Empress Direct	23.0±1.0	0.58±0.04
Tetric EvoCeram	13.4±0.6	0.29±0.03
Amaris	10.0±0.2	0.18±0.04
GrandioSo	16.0±0.3	0.31±0.04
Gradia Direct X	10.3±0.3	0.27±0.03
GC Kalore	10.4±0.3	0.25±0.01
Ice	17.4±0.5	0.44±0.03
N'Durance	38.3±1.1	0.87±0.06
Clearfil AP-X	22.0±0.3	0.54±0.05
Clearfil Majesty Esthetic	24.0±0.9	0.51±0.02
Synergy D6	11.2±0.3	0.30±0.02
Esthet-X HD	16.5±1.2	0.57±0.03
TPH3	21.1±1.2	0.59±0.04
Ceram-X	29.6±1.2	0.79±0.03
Artiste Nano	15.6±0.4	0.47±0.05
Simile	14.7±1.1	0.34±0.02
Herculite Ultra	11.5±0.4	0.34±0.02

Units are normalized to the dpph signal intensity. Sem: standard error of the mean.

For experimental resins (table 4), a large variation of intensity was observed among the pure monomers, Bis-GMA giving the strongest signal of 106 n.u., whereas Bis-EMA-15 gave only an intensity of 0.4 n.u. (median  = 63.2 n.u.) Compositions made with various proportions of these monomers gave a higher signal when the content in Bis-GMA or UDMA was high. Nevertheless, there was no direct and simple relation between the composition and the signal intensity, suggesting that the rate of radical recombination or termination was different in the monomer mixtures than in the pure resins.

**Table 4:** Normalized intensities of EPR signal recorded in experimental resins 5 minutes after polymerization (X-band) and 65 minutes after polymerization (L-band). Units are normalized to the signal intensity of dpph.

Experimental resins	Intensity 5 min X-Band Normalized units ± sem (n = 5)	Intensity 65 min L-Band Normalized units ± sem (n = 5)
G 100%	106.9±5.3	1.1±0.1
T 100%	3.8±0.6	nd
U 100%	60.5±1.4	0.70±0.05
E-15 100%	0.4±0.1	nd
E-2 100%	41.3±0.7	0.57±0.03
G/T 0.3582/0.6418	80. 8±2.0	1.56±0.02
G/T 0.5659/0.4341	87.5±3.3	1.57±0.04
G/U 0.3582/0.6418	71.9±2.7	1.09±0.02
G/U 0.5659/0.4341	67.4±3.1	1.17±0.05
G/E-15 0.3582/0.6418	5.5±0.2	0.25±0.02
G/E-15 0.5659/0.4341	37.3±0.8	0.69±0.03
G/T/U/E-15 0.5660/0.2340/0.1/0.1	63.2±1.1	1.10±0.03
G/T/U/E-15 0.5660/0.1340/0.15/0.15	77.7±1.3	1.43±0.02
G/U/E-15 0.5660/0.2170/0.2170	74.7±3.1	0.80±0.05

G: Bis-GMA, T: TEGDMA, U: UDMA, E-15: Bis-EMA (15-ethoxy/phenol), E-2: Bis-EMA (2-ethoxy/phenol). Sem: standard error of the mean. Nd: not detectable.

### Characterization of the signal decay kinetics

**Figure 4:**
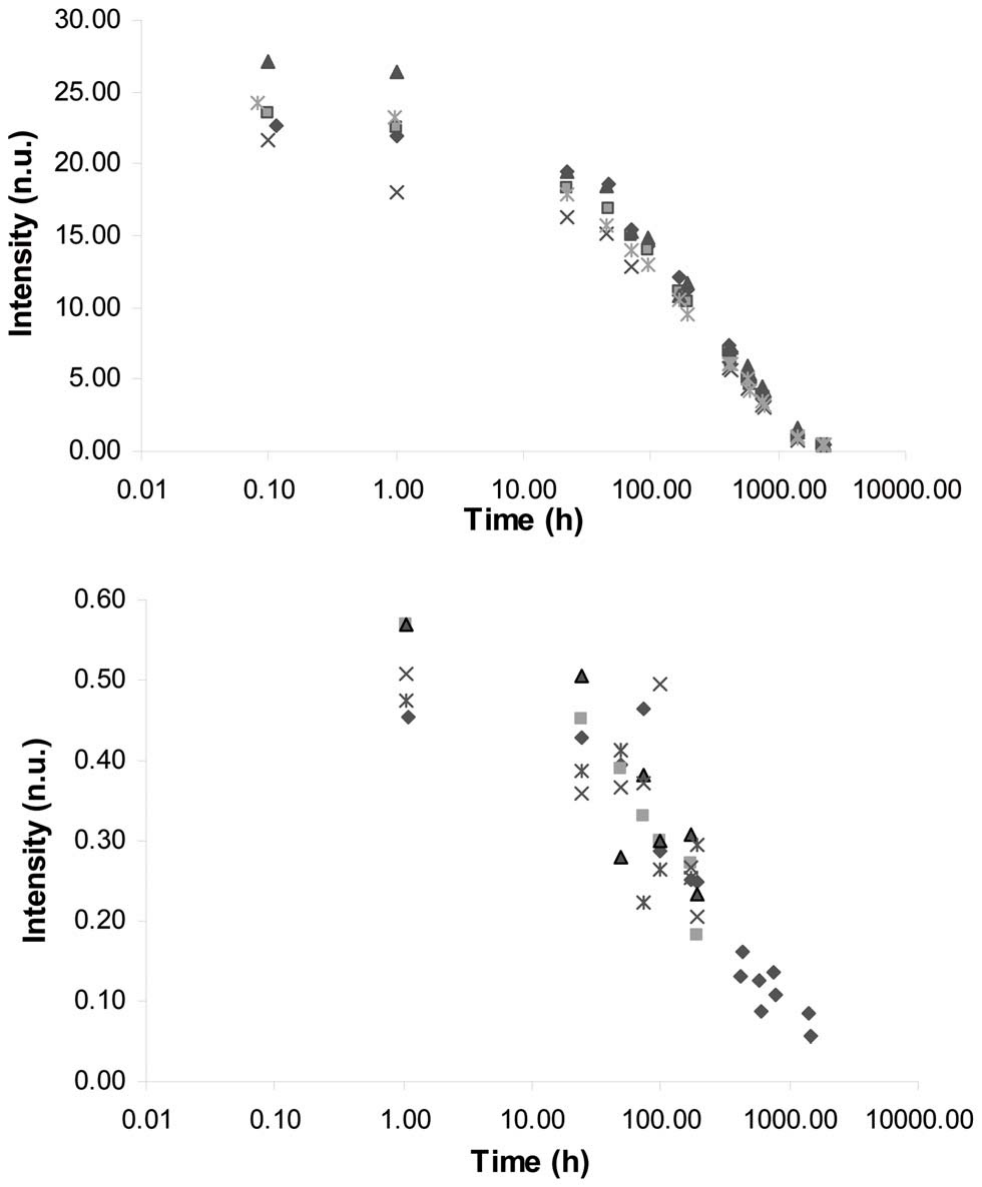
Decay curves recorded in X-band (top) and L-band (bottom) for the Clearfil Majesty Esthetic composite. Decay curves were measured for 5 samples.

**Figure 5:**
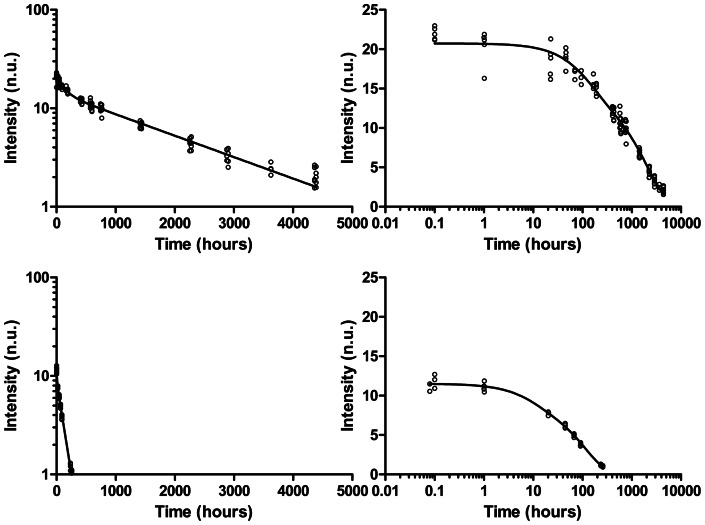
Bi-exponential fitting of the decay curve recorded in X-band for typical composites: Clearfil AP-X (top) and Herculite Ultra (bottom).

Because this type of signal had been described as decaying with time for some composites [25], we investigated the full decay curve over a six months period. A good reproducibility of the signal was observed both in X-band and in L-band (Fig. 4). For most of the composites, the curve could be fitted with a bi-exponential decay model (Fig. 5). For three composites however (Tetric EvoCeram, Amaris and GC Kalore, table 5), the decay of the second component of the model was so slow that it was not statistically significant and consequently reduced to a mono-exponential model.

**Table 5:** Decay kinetics parameters for commercial resins using a bi-exponential model.

Commercial composites	1st compartment T_1/2_ (h) + CI 95%	2^nd^ compartment T_1/2_ (h) + CI 95%
Synergy D6	1.0 (0.8–1.6)	171 (159–186)
N'Durance	1.2 (1.0–1.7)	51 (47–57)
TPH3	1.5 (0.8–13.0)	332 (302–368)
Herculite Ultra	7.1 (3.9–41.7)	80 (71–93)
Simile	10 (6–47)	239 (210–277)
Esthet-X HD	10(6–52)	210 (186–241)
Ice	12 (8–27)	261 (237–291)
Artiste Nano	14 (10–25)	257 (228–293)
Ceram-X	27 (19–45)	273 (241–315)
Clearfil Majesty Esthetic	27 (19–47)	355 (312–411)
IPS Empress Direct	40 (32–54)	350 (234–692)
Filtek Supreme Ultra	43 (38–50)	640 (544–779)
Venus Diamond	74 (63–89)	1345 (1102–1727)
GrandioSo	80 (69–96)	1199 (800–2387)
GC Kalore	94 (64–184)	ns
Clearfil AP-X	95 (71–143)	1382 (1253–1542)
Tetric EvoCeram	134 (76–602)	ns
Amaris	187 (130–331)	ns
Gradia Direct X	218 (142–473)	4601 (168–∞)

Half-lives are in hours with confidence interval at 95%. Ns: non significant.

Regression was performed on 5 samples.

The majority of the commercial composites showed a rapid decay rate for the first component, with a half-life below 48 h (table 5). Five resins (GC Kalore, Clearfil AP-X, Tetric EvoCeram, Amaris, Gradia Direct X) showed a particularly slow first phase decay with a half-life above 100 h.

When applicable, the decay in the second compartment was much slower than in the first one.

Four resins (Grandioso, Venus Diamond, Clearfil AP-X, Gradia Direct X) showed an extremely slow decay with a half-life above 1000 hours, the other ones showing a somewhat faster decay, with half-lives between 200 and 650 hours.

For experimental resins, a bi-exponential model could also be applied, with the exception of the pure monomer Bis-EMA-15, for which the initial signal intensity was low and no intensity decay was observed over the time period considered. Half-lives observed for pure resins ranged from 36 h to 239 h (table 6). Interestingly, the half-lives observed for the various compositions of monomers were systematically smaller than what would be expected if they merely reflected the decay of the individual monomers. This could suggest a better mobility of molecules so that recombination or termination happened faster. In experimental compositions, high molecular weight and/or stiff monomers were added to low molecular weight and/or flexible monomers. The latter lead to a higher molecular mobility in the vitrified system, thereby favouring bimolecular radical termination through a reaction-diffusion-controlled termination mechanism, which dominates the most rapid radical decrease during the first few hours after photopolymerization [26].

**Table 6:** Decay kinetics parameters for experimental compositions using a bi-exponential model.

Experimental resins	1st compartment T_1/2_ (h) + CI 95%	2^nd^ compartment T_1/2_ (h) + CI 95%
G 100%	99 (80–127)	1761 (1448–2247)
T 100%	138 (120–164)	2164 (129–∞)
U 100%	239 (182–349)	2866 (2579–3226)
E-15 100%	Zero order kinetics	na
E-2 100%	36 (30–44)	234 (149–538)
G/T 0.3582/0.6418	39 (34–45)	939 (865–1027)
G/T 0.5659/0.4341	67 (56–85)	1047 (920–1216)
G/U 0.3582/0.6418	88 (69–121)	1749 (1579–1960)
G/U 0.5659/0.4341	62 (52–77)	1288 (1193–1400)
G/E-15 0.3582/0.6418	1.0 (0.8–1.1)	14009 (3170–∞)
G/E-15 0.5659/0.4341	10 (7–15)	107 (89–135)
G/T/U/E-15 0.5660/0.2340/0.1/0.1	75 (64–92)	958 (806–1180)
G/T/U/E-15 0.5660/0.1340/0.15/0.15	52 (45–61)	570 (507–650)
G/U/E-15 0.5660/0.2170/0.2170	70 (59–87)	545 (424–764)

Half-lives are in hours with Confidence interval at 95%. Na: not applicable. G: Bis-GMA, T: TEGDMA, U: UDMA, E-15: Bis-EMA (15-ethoxy/phenol), E-2: Bis-EMA (2-ethoxy/phenol).

Regression was performed on 5 samples.

From the operations standpoint, it was important to determine the maximum timeframe in which an EPR signal could be detected, and potentially interfere with the dosimetric signal from the enamel. As expected from the various levels of initial intensity, and the various decay kinetics, the limit of detection varied a lot from composite to composite (table 7), or from experimental resin to another (table 8). Overall, six months after polymerization, the typical 9 lines EPR signal could not be detected in X-band in any of the commercial composites tested, except for the Clearfil AP-X. In L-band at 1.2 GHz, which is about 100 times less sensitive, this period was reduced to 18 days for the resin giving the longer lasting signal.

**Table 7:** Detection limit of the EPR signal for commercial composites, expressed as the time period needed for disappearance of the signal under the measurements conditions.

Commercial composites	X-band detection threshold	L-band detection threshold
Filtek Supreme Ultra	5 months	16 days
Venus Diamond	5 months	1 day
IPS Empress Direct	2 months*	4 days
Tetric EvoCeram	2 months*	2 days
Amaris	2 months	0 day
GrandioSo	2 months*	1 day
Gradia Direct X	3 months	1 day
GC Kalore	2 months	1 day
Ice	3 months	8 days
N'Durance	17 days	3 days
Clearfil AP-X	>6 months	18 days
Clearfil Majesty Esthetic	3 months	8 days
Synergy D6	2 months	1 day
Esthet-X HD	2 months	3 days
TPH3	3 months	4 days
Ceram-X	2 months*	10 days
Artiste Nano	2 months	4 days
Simile	2 months	3 days
Herculite Ultra	18 days	2 days

The mass of the sample was 30 mg, a typical medium size restoration on an incisor.

* detection threshold for the nine-lines signal. See table 8 for nitroxide-like signal threshold.

**Table 8:** Detection limit of the EPR signal for experimental resins and mixtures, expressed as the time period needed for disappearance of the signal under the measurements conditions.

Experimental resins	X-band detection threshold	L-band detection threshold
G 100%	>6 months	>5 months
T 100%	2 months	0 day
U 100%	>6 months	>5 months
E-15 100%	1 day	0 day
E-2 100%	1 month	16 days
G/T 0.3582/0.6418	>6 months	3 months
G/T 0.5659/0.4341	>6 months	3 months
G/U 0.3582/0.6418	>6 months	3 months
G/U 0.5659/0.4341	>6 months	3 months
G/E-15 0.3582/0.6418	2 days	1 hour
G/E-15 0.5659/0.4341	1 month	3 days
G/T/U/E-15 0.5660/0.2340/0.1/0.1	>6 months	3 months
G/T/U/E-15 0.5660/0.1340/0.15/0.15	>6 months	3 months
G/U/E-15 0.5660/0.2170/0.2170	5 months	3 months

The mass of the sample was 30 mg, a typical medium size restoration on an incisor. G: Bis-GMA, T: TEGDMA, U: UDMA, E-15: Bis-EMA (15-ethoxy/phenol), E-2: Bis-EMA (2-ethoxy/phenol).

**Figure 6:**
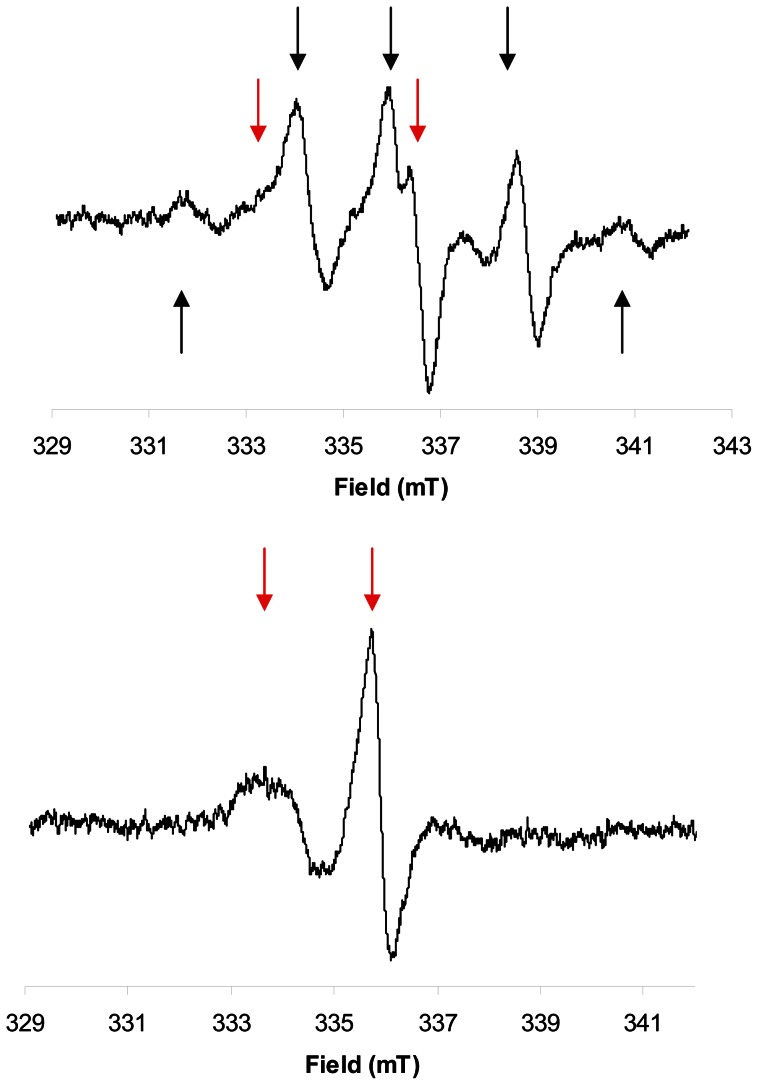
EPR signal observed in X-band for the GrandioSo composite, 1 month after polymerization (top) and 3 months after polymerization (bottom). Black arrows show lines of the documented EPR spectrum of resins. Red arrows show lines from the atypical component of the spectrum.

Nevertheless, it must be mentioned that in some cases (IPS Empress Direct, Tetric EvoCeram, GrandioSo, Ceram-X), an atypical signal could be observed, in X-band, when the nine lines signal had sufficiently decayed (Fig. 6 & table 9). This signal remained stable during the measuring period and presented a hyperfine splitting which is compatible with the spectrum of an immobilized nitroxide. It was observed reproducibly (n = 5) only in the mentioned composites. Contamination (cavity, tubes etc.) was excluded by control experiments. Because this type of nitroxide-like signal was not observed in the experimental resins, which are lacking additives found in commercial ones, such as pigments etc., it is likely that a cross radical reaction occurred between resin radicals and one of these additives present in low concentration, but of undisclosed structure. In L-band, since the sensitivity is lower than in X-band, no signal was detectable as early as one day after polymerization for some composites (table 7).

**Table 9:** Detection limit of composites showing the atypical nitroxide-like signal.

Commercial composites	X-band detection threshold Nitroxide-like signal
IPS Empress Direct	>6 months
Tetric EvoCeram	>6 months
GrandioSo	>6 months
Ceram-X	>6 months

## Discussion

This work is the first study providing an extensive characterization of the EPR signal arising from dental composites widely used for teeth restorations.

We have demonstrated that a strong EPR signal was detected in all the composites immediately after initiation of the photopolymerization process, and that this signal could dramatically affect the dosimetric signal from the enamel, because of its spectral position (frequency of resonance) similar to that of the dosimetric signal, and because of its intensity.

Fortunately, the resin signal was rapidly decaying, so that the signal had completely disappeared within six months. In L-band, from an operations standpoint, the signal was negligible as early as 18 days after the polymerization. It must nevertheless be mentioned that L-band spectrometry using surface resonator is very sensitive to the geometrical factor. The shape of the loop, as well as the position of the sample in the loop, can significantly affect the recorded intensity of the signal. Commercial instruments are most probably less sensitive than those developed for detection of the dosimetric signal directly in the mouth. The results obtained in this study should not be transposed as such to other spectrometers, but could easily be confirmed for each type of resonator based on the data here provided.

The results obtained for experimental compositions, composed of the most common monomers found in commercial resins, should help in evaluating the possible influence of each resin on the dosimetric signal.

Some important aspects, related to other influences that resins could have on the dosimetric signal, remain to be fully investigated in order to complete this part of the validation of the method.

A first issue is the possible EPR signal induced in the resin when exposed to ionizing radiation. Dental composites contain a large fraction of inorganic filler (roughly 50 to 80% wt), mainly glass, which are known to give an EPR signal when exposed to ionising radiations. Free radicals are also likely to be induced in the organic matrix, where they can remain stable for an undetermined period. Indeed, in a limited set of experiments, we observed a broad multiline EPR signal in some composites exposed, at this stage, to high doses. A complete screening of the composites described in this study and a characterisation of this broad signal would be required to evaluate its possible influence on the dosimetric signal.

A second issue is the underestimation of the dose if measurements are performed on a restored tooth. If the restoration is old enough, no EPR signal from the resins will directly interfere. Nevertheless, the presence of resin in a tooth will decrease the amount of enamel measured. As the intensity of the EPR signal is also proportional to the quantity of enamel, the intensity of the EPR dosimetric signal in the enamel will be less intense than an intact tooth. Because the *in vivo* dosimetry uses calibration curve established on intact tooth, the dose can be therefore underestimated, depending on the amount of resin in the measured tooth.

Another issue is the influence of the light emitted by the dental curing light on the dosimetric signal. UV radiations are known to induce an EPR signal in enamel, resembling the dosimetric one [27], [28]. Since curing lights used by dentists emit in the blue and near-UV wavelengths, they might also generate a signal. Nevertheless, the light used in this study only emits in the visible part of the spectrum (380–530 nm), with two peaks, one at 410 nm (irradiance  = 302 mW/cm^2^) and the other at 464 nm (irradiance  = 1153 mW/cm^2^). Moreover, the illumination time is very short and does not usually exceed 30 s. Consequently the influence of the dental light on the dosimetric signal should be negligible, but should be checked for other dental lights used in routine clinical practice.

Overall, this study demonstrates that the EPR signal arising from composites used for tooth restoration should not affect by itself the dosimetric signal, in most cases. Caution should be paid to recent restorations, or to restorations performed with composites known for giving a long lasting atypical EPR signal. In these circumstances, external lines might not be detected, while the central line would still affect the dosimetric signal. This possible contribution should be evaluated for doses close to the detection limit of CO_2_^−^ radicals in L-band.

## References

[1] Coleman CN, Hrdina C, Bader JL, Norwood A, Hayhurst R, et al (2009) Medical response to a radiologic/nuclear event: integrated plan from the Office of the Assistant Secretary for Preparedness and Response, Department of Health and Human Services. Ann Emerg Med 53: 213–222. 18387707 10.1016/j.annemergmed.2007.12.021

[2] Bushberg JT, Kroger LA, Hartman MB, Leidholdt EM Jr, Miller KL, et al (2007) Nuclear/radiological terrorism: emergency department management of radiation casualties. J Emerg Med 32: 71–85. 17239736 10.1016/j.jemermed.2006.05.034

[3] Goransson Nyberg A, Stricklin D, Sellstrom A (2011) Mass casualties and health care following the release of toxic chemicals or radioactive material-contribution of modern biotechnology. Int J Environ Res Public Health 8: 4521–4549. 22408587 10.3390/ijerph8124521 PMC3290976

[4] Koenig KL, Goans RE, Hatchett RJ, Mettler FA Jr, Schumacher TA, et al (2005) Medical treatment of radiological casualties: current concepts. Ann Emerg Med 45: 643–652. 15940101 10.1016/j.annemergmed.2005.01.020

[5] Anno GH, Baum SJ, Withers HR, Young RW (1989) Symptomatology of acute radiation effects in humans after exposure to doses of 0.5–30 Gy. Health Phys 56: 821–838. 2722506 10.1097/00004032-198906000-00001

[6] Anno GH, Young RW, Bloom RM, Mercier JR (2003) Dose response relationships for acute ionizing-radiation lethality. Health Phys 84: 565–575. 12747475 10.1097/00004032-200305000-00001

[7] Grace MB, Moyer BR, Prasher J, Cliffer KD, Ramakrishnan N, et al (2010) Rapid radiation dose assessment for radiological public health emergencies: roles of NIAID and BARDA. Health Phys 98: 172–178. 20065680 10.1097/01.HP.0000348001.60905.c0

[8] Hafer N, Cassatt D, Dicarlo A, Ramakrishnan N, Kaminski J, et al (2010) NIAID/NIH radiation/nuclear medical countermeasures product research and development program. Health Phys 98: 903–905. 20445403 10.1097/HP.0b013e3181bbc4df

[9] Blakely WF, Carr Z, Chu MC, Dayal-Drager R, Fujimoto K, et al (2009) WHO 1st consultation on the development of a global biodosimetry laboratories network for radiation emergencies (BioDoseNet). Radiat Res 171: 127–139. 19138057 10.1667/RR1549.1

[10] Kulka U, Ainsbury L, Atkinson M, Barquinero JF, Barrios L, et al (2012) Realising the European Network of Biodosimetry (RENEB). Radiat Prot Dosimetry 151: 621–625. 22923244 10.1093/rpd/ncs157

[11] Wojcik A, Lloyd D, Romm H, Roy L (2010) Biological dosimetry for triage of casualties in a large-scale radiological emergency: capacity of the EU member states. Radiat Prot Dosimetry 138: 397–401. 19951985 10.1093/rpd/ncp279

[12] Leonard A, Rueff J, Gerber GB, Leonard ED (2005) Usefulness and limits of biological dosimetry based on cytogenetic methods. Radiation Protection Dosimetry 115: 448–454. 16381765 10.1093/rpd/nci061

[13] Ainsbury EA, Bakhanova E, Barquinero JF, Brai M, Chumak V, et al (2011) Review of retrospective dosimetry techniques for external ionising radiation exposures. Radiat Prot Dosimetry 147: 573–592. 21183550 10.1093/rpd/ncq499

[14] Fattibene P, Callens F (2010) EPR dosimetry with tooth enamel: A review. Appl Radiat Isot 68: 2033–2116. 20599388 10.1016/j.apradiso.2010.05.016

[15] Cole T, Silver AH (1963) Production of hydrogen atoms in teeth by X-irradiation. Nature 200: 700–701. 14109974 10.1038/200700a0

[16] Swartz HM (1965) Long-Lived Electron Spin Resonances in Rats Irradiated at Room Temperature. Radiation Research 24: 579–586. 14275318

[17] Williams BB, Dong R, Nicolalde RJ, Matthews TP, Gladstone DJ, et al (2011) Physically-based biodosimetry using in vivo EPR of teeth in patients undergoing total body irradiation. Int J Radiat Biol 87: 766–775. 21696339 10.3109/09553002.2011.583316 PMC4086327

[18] Swartz HM, Flood AB, Williams BB, Dong R, Swarts SG, et al (2012) Electron paramagnetic resonance dosimetry for a large-scale radiation incident. Health Phys 103: 255–267. 22850230 10.1097/HP.0b013e3182588d92 PMC3649772

[19] Gomez JA, Marques T, Kinoshita A, Belmonte G, Nicolucci P, et al (2011) Influence of dental restorative materials on ESR biodosimetry in tooth enamel. Radiat Res 176: 259–263. 21631287 10.1667/rr2503.1

[20] Cramer NB, Stansbury JW, Bowman CN (2011) Recent advances and developments in composite dental restorative materials. J Dent Res 90: 402–416. 20924063 10.1177/0022034510381263 PMC3144137

[21] Leprince J, Palin WM, Mullier T, Devaux J, Vreven J, et al (2010) Investigating filler morphology and mechanical properties of new low-shrinkage resin composite types. J Oral Rehabil 37: 364–376. 20202096 10.1111/j.1365-2842.2010.02066.x

[22] Leprince JG, Palin WM, Hadis MA, Devaux J, Leloup G (2012) Progress in dimethacrylate-based dental composite technology and curing efficiency. Dent Mater. 10.1016/j.dental.2012.11.005 23199807

[23] Truffier-Boutry D, Gallez XA, Demoustier-Champagne S, Devaux J, Mestdagh M, et al (2003) Identification of free radicals trapped in solid methacrylated resins. Journal of Polymer Science Part A: Polymer Chemistry 41: 1691–1699.

[24] Sideridou I, Tserki V, Papanastasiou G (2003) Study of water sorption, solubility and modulus of elasticity of light-cured dimethacrylate-based dental resins. Biomaterials 24: 655–665. 12437960 10.1016/s0142-9612(02)00380-0

[25] Leprince J, Lamblin G, Truffier-Boutry D, Demoustier-Champagne S, Devaux J, et al (2009) Kinetic study of free radicals trapped in dental resins stored in different environments. Acta Biomaterialia 5: 2518–2524. 19500595 10.1016/j.actbio.2009.04.034

[26] Leprince JG, Lamblin G, Devaux J, Dewaele M, Mestdagh M, et al (2010) Irradiation Modes' Impact on Radical Entrapment in Photoactive Resins. Journal of Dental Research 89: 1494–1498. 20940363 10.1177/0022034510384624

[27] Jiao L, Takada J, Endo S, Tanaka K, Zhang W, et al (2007) Effects of sunlight exposure on the human tooth enamel ESR spectra used for dose reconstruction. J Radiat Res 48: 21–29. 17159329 10.1269/jrr.0616

[28] Sholom S, Desrosiers M, Chumak V, Luckyanov N, Simon SL, et al (2010) UV effects in tooth enamel and their possible application in EPR dosimetry with front teeth. Health Phys 98: 360–368. 20065706 10.1097/01.HP.0000348002.69740.bd PMC2808200

